# 291. A Longitudinal Study of The Effect of Fecal Microbiota Transplantation on *Klebsiella pneumoniae* and its Bacteriophages in a Patient with Long-Term Colonization

**DOI:** 10.1093/ofid/ofad500.363

**Published:** 2023-11-27

**Authors:** Joshua Manuel, Sarah Forsstrom, Brandon A Berryhill, Kylie Burke, Andrew Smith, Michael H Woodworth, Bruce Levin

**Affiliations:** Emory University, Atlanta, Georgia; Emory University, Atlanta, Georgia; Emory University, Atlanta, Georgia; Emory University, Atlanta, Georgia; Emory University, Atlanta, Georgia; Emory University, Atlanta, Georgia; Emory University, Atlanta, Georgia

## Abstract

**Background:**

Live biotherapeutic products such as fecal microbiota transplantation (FMT) show promise as therapies for conditions associated with gut microbiome composition and function, including colonization and recurrent infection with multidrug resistant organisms (MDRO). Although FMTs can reduce colonization with MDROs, much is still unknown about the mechanisms responsible for successful treatment with FMTs. The observation that bacteriophages (phages) are ubiquitous in all microbial communities including FMT doses and are being developed as therapies for MDROs suggests that phages contribute to FMT efficacy. We present results of a case study employing experimental and metagenomic analyses to test the hypothesis that phages contribute to the clinical efficacy of FMTs.

A patient with recurrent urinary tract infections (UTI) with a multidrug resistant Klebsiella pneumoniae was treated with an FMT delivered by retention enema as part of a recent clinical trial (NCT02922816). Metagenomic analyses showed that this patient had baseline K. pneumoniae intestinal domination with relative abundance of 90%. At Day 2 post FMT, the relative abundance of K. pneumoniae was 0.5% and follow-up stool cultures with Klebsiella selective and differential media were negative.

**Methods:**

We competitively aligned reads from the stool metagenomes to the gut phage database using bowtie2 and inStrain. We isolated *K. pneumoniae* and phages from the donor and patient samples and bacteria susceptibility.

Methods For K. pneumoniae/Phage Isolation and Metagenomic Analysis

**Figure:**
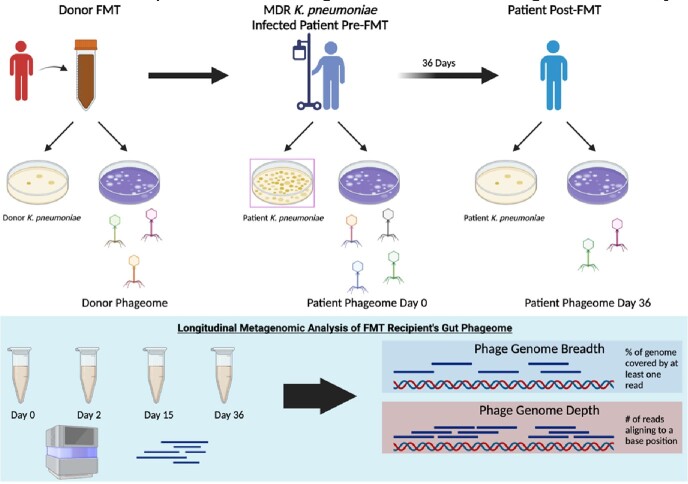

**Results:**

We found 76 potential *K. pneumoniae* phages in the patient before treatment, of which 58 have a breadth of coverage > 0.10. Among these, 39/58 (67%) are known to be temperate and are able to incorporate in the host bacterial genomes as prophages.

Breadth of Coverage of K. pneumoniae Phages in the Patient Prior to FMT

**Figure:**
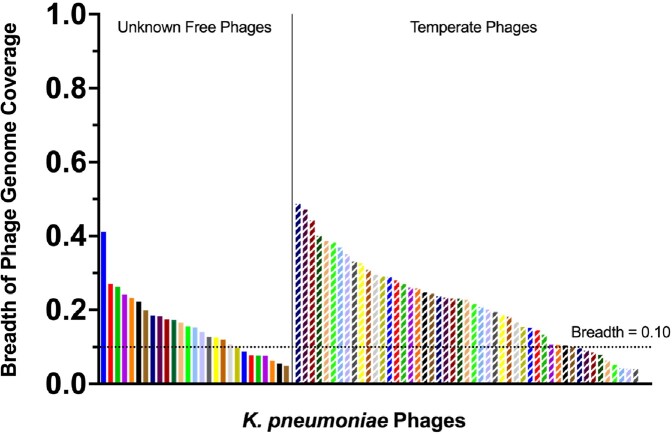

Solid bars represent unknown free phages identified in the sample. Dashed bars represent phages that are known to be temperate found in the patients metagenome.

**Conclusion:**

As resistance to host phages quickly emerges, we postulated that phages in the FMT dose contribute to the elimination of *K. pneumoniae* in this patient. To test the hypothesis, we follow the changes in relative densities of gut *K. pneumoniae* phages with metagenomic coverage analyses in longitudinally collected fecal samples post FMT. To further test this hypothesis, we isolated phages and bacteria from the FMT and sequential fecal samples from the patient and tested the susceptibility of the *K. pneumoniae* to the phages from both the FMT dose and the patient.

**Disclosures:**

**All Authors**: No reported disclosures

